# Characterization of Immune-Based Molecular Subtypes and Prognostic Model in Prostate Adenocarcinoma

**DOI:** 10.3390/genes13061087

**Published:** 2022-06-18

**Authors:** Li Guo, Yihao Kang, Daoliang Xia, Yujie Ren, Xueni Yang, Yangyang Xiang, Lihua Tang, Dekang Ren, Jiafeng Yu, Jun Wang, Tingming Liang

**Affiliations:** 1 Department of Bioinformatics, Smart Health Big Data Analysis and Location Services Engineering Lab of Jiangsu Province, School of Geographic and Biologic Information, Nanjing University of Posts and Telecommunications, Nanjing 210023, China; lguo@njupt.edu.cn (L.G.); 1219012909@njupt.edu.cn (Y.K.); 1220013736@njupt.edu.cn (D.X.); 1021173610@njupt.edu.cn (Y.R.); b18080407@njupt.edu.cn (X.Y.); 1221014238@njupt.edu.cn (Y.X.); tanglh@njupt.edu.cn (L.T.); 1021173609@njupt.edu.cn (D.R.); 2 Shandong Provincial Key Laboratory of Biophysics, Institute of Biophysics, Dezhou University, Dezhou 253023, China; jfyu1979@126.com; 3 Jiangsu Key Laboratory for Molecular and Medical Biotechnology, School of Life Science, Nanjing Normal University, Nanjing 210023, China

**Keywords:** prostate adenocarcinoma (PRAD), immune-based, molecular subtypes, prognostic model

## Abstract

Prostate adenocarcinoma (PRAD), also named prostate cancer, the most common visceral malignancy, is diagnosed in male individuals. Herein, in order to obtain immune-based subtypes, we performed an integrative analysis to characterize molecular subtypes based on immune-related genes, and further discuss the potential features and differences between identified subtypes. Simultaneously, we also construct an immune-based risk model to assess cancer prognosis. Our findings showed that the two subtypes, C1 and C2, could be characterized, and the two subtypes showed different characteristics that could clearly describe the heterogeneity of immune microenvironments. The C2 subtype presented a better survival rate than that in the C1 subtype. Further, we constructed an immune-based prognostic model based on four screened abnormally expressed genes, and they were selected as predictors of the robust prognostic model (AUC = 0.968). Our studies provide reference for characterization of molecular subtypes and immunotherapeutic agents against prostate cancer, and the developed robust and useful immune-based prognostic model can contribute to cancer prognosis and provide reference for the individualized treatment plan and health resource utilization. These findings further promote the development and application of precision medicine in prostate cancer.

## 1. Introduction

Prostate adenocarcinoma (PRAD), also termed prostate cancer, is the second most commonly diagnosed malignant cancer in males, especially for elderly men over 65 years of age [1], which has been a leading cause of cancer-related morbidity and mortality [2,3]. Some patients (approximately one third of patients) may experience disease progression and develop metastases, most commonly to bone and soft tissues (such as liver and lung) [4,5]. It is still a significant global heath burden in the future, because long-term survival and advanced stages still need be improved [6]. A healthy immune system is necessary for controlling many malignant diseases, and immune suppression in cancer may contribute to progression [7]. Recent studies based on immunology and cancer biology have provided the new directions to the immune-based therapy of prostate cancer, especially focusing on passive and active immunotherapies [8,9]. Metabolic inhibitors, including the anti-metabolite class of chemotherapy, have been used in cancer therapies for many years [10,11], but no metabolic inhibitors are approved for use in PRAD. It is necessary to characterize and verify novel biomarkers for diagnosis and treatment, and to develop novel targeted therapies and/or neoadjuvant therapies. 

Major changes in gene transcription and metabolic signaling contribute to tumorigenesis [12]. Many studies have shown that some genes, also including non-coding RNAs (ncRNAs), largely contribute to tumorigenesis. For example, rAAV-based PTEN/CDKN1B delivery is promising for the development of novel therapeutics for PRAD because of its potential contribution [13], miR-338-3p may be a possible predictor of poor prognosis [14], some lncRNAs may be novel biomarkers in the diagnosis and prognosis of PRAD [15], and the lncRNA H19 regulates tumor plasticity in neuroendocrine prostate cancer [16]. These findings indicate the potential biological roles of some crucial genes in the occurrence and development of cancer, and further studies can be performed based on the potential crucial genes to explore the biomarkers in cancer diagnose and prognosis and even to characterize molecular classification. Molecular classification has been an important topic in clinical management because of the heterogeneity caused primarily by the molecular and genetic characteristics of cancer cells, which actually determine the aggressiveness and sensitivity of further treatment. An integrative analysis based on multi-omics data characterizes molecular subtypes that can guide the androgen receptor signaling inhibitor treatment of prostate cancer [17], which can contribute to targeted therapy in precision medicine. 

Based on the significant curative effect of immunotherapy in cancers, in order to comprehensively understand the detailed immune-based molecular classification, we performed an integrative analysis to screen candidate crucial immune-related genes to explore the molecular subtypes associated with PRAD. We finally obtained two immune-related molecular subtypes that showed potential difference in immune characteristics, which definitely describe the heterogeneity of diverse immune microenvironments in different patients. Further, we also constructed a prognostic model using four dysregulated immune-related genes that could provide a robust and useful model for individualized treatment. Our findings provide a powerful data basis for further immunotherapy of PRAD that will contribute to the application of precision medicine. 

## 2. Materials and methods

### 2.1. Data Resource

A total of 551 samples of PRAD, including 499 tumor and 52 normal samples, were retrieved from the cancer genome atlas (TCGA, https://tcga-data.nci.nih.gov/tcga/ (accessed on 21 May 2021)) via UCSC Xena database [18]. Some samples were removed before further analysis if there were many missing clinical values. A total of 28 gene sets, containing 782 genes, were first collected from immune cell types [19]. In order to further understand the detailed expression patterns for screened key genes, a pan-cancer analysis was also performed in diverse cancers that were obtained from TCGA. Furthermore, a total of 1,793 immune-related genes were obtained from ImmPort database [20,21]. 

### 2.2. Characterization of Immune-Based Subtypes

Based on 782 immune-related genes, single sample gene set enrichment analysis (ssGSEA) was first analyzed in each sample using GSVA package [22]. Consensus clustering analysis of ssGSEA score was performed with ConsensusClusterPlus package [23] using k-means clustering algorithm with 50 iterations (each analysis used 80% of samples). Then, the best cluster number was selected according to the cumulative distribution function (CDF) curve, which was further validated using t-SNE method. 

### 2.3. Evaluation of Immune Characteristics in Subtypes

In order to understand the potential difference of immune characteristics between identified subtypes, two features, including stromal signature and immune signature, were screened using ESTIMATE [24]. Scores of stromal and immune signatures were estimated using ssGSEA analysis, and the ESTIMATE score was finally obtained to assess tumor purity from the two scores. Then, the potential differences of related immune characteristics between different subtypes were estimated using Kruskal–Wallis test. 

### 2.4. Functional Enrichment Analysis

For the screened subtypes in PRAD, functional enrichment analysis was performed to understand their potential biological function, especially the potential contributions in the occurrence and development of cancer. Firstly, differentially expressed genes in the C2 subtype (screened based on the C1 subtype) were performed gene set enrichment analysis (GSEA), and FDR < 0.05 was considered with a significant difference. 

### 2.5. The Potential Differences of Immune and Chemical Response Prediction

To explore the potential differences of immunotherapy and chemotherapy between the two subtypes, the clinical responses of immune subtypes were predicted and analyzed. According to the public available pharmacogenomic database, Genomics of Drug Sensitivity in Cancer (GDSC) [25], the chemotherapy response in each sample was predicted. Simultaneously, six commonly used drugs were specifically selected, including Bortezomib, Paclitaxel, Epirubicin, Vincristine, Gemcitabine, and Vorinostat, to carry out drug response analysis using oncoPredict package [26]. 

### 2.6. Analysis of Differentially Expressed Immune-Related Genes

To obtain differentially expressed immune-related genes associated with PRAD, limma package [27] and DESeq2 [28] were used to perform differential expression analysis. Genes with |log_2_FC| > 1.5 and padj < 0.05 were considered abnormally expressed genes that were performed further analysis. 

### 2.7. Survival Analysis and Cox Regression Analysis

Based on the screened differentially expressed immune-related genes, Cox regression analysis was used to estimate the correlation of gene expression level and the overall survival (OS) in patient using survival package using univariate cox regression analysis. The candidate crucial genes associated with cancer prognosis were screened based on Walk test (*p* < 0.05 was considered with statistical difference). Then, the candidate genes were performed to Cox multivariate regression analysis to construct several Cox regression models, and the dominant model was identified according to Akaike information criterion (AIC) value. Genes in the finally identified model were critical genes associated with PRAD, which were used to estimate the risk score according to the following formula (1):

(1) $$\mathrm{Risk}\mathrm{score}=\sum_{i=1}^{N}({\mathrm{Exp}}_{i}\times C_{i})$$

in which *N* was the number of critical genes associated with cancer prognosis, Exp_i_ indicated expression level of the gene, *C_i_* was estimated regression coefficient of the gene. 

According to the developed prognostic model, risk score was assessed in each patient, and high-risk and low-risk groups were divided according to risk scores. Kaplan–Meier method [29] was then used to perform survival analysis. Further, to validate accuracy and effectiveness of the developed prognostic model, the receiver-operating characteristics (ROC) was used to compare the diagnostic power according to the area under the ROC curve (AUC). 

### 2.8. Statistical Analysis

Unpaired *t* test, chi-square test, Wilcoxon rank sum test and trend test were performed to validate the potential statistical difference between groups. A Pearson or Spearman correlation coefficient was used to calculate the expression correlations among genes. All statistical analysis was performed using R programming language (version 4.0.5).

## 3. Results

### 3.1. Two Molecular Subtypes Identified using Immune-Based Genes

**Figure 1:**
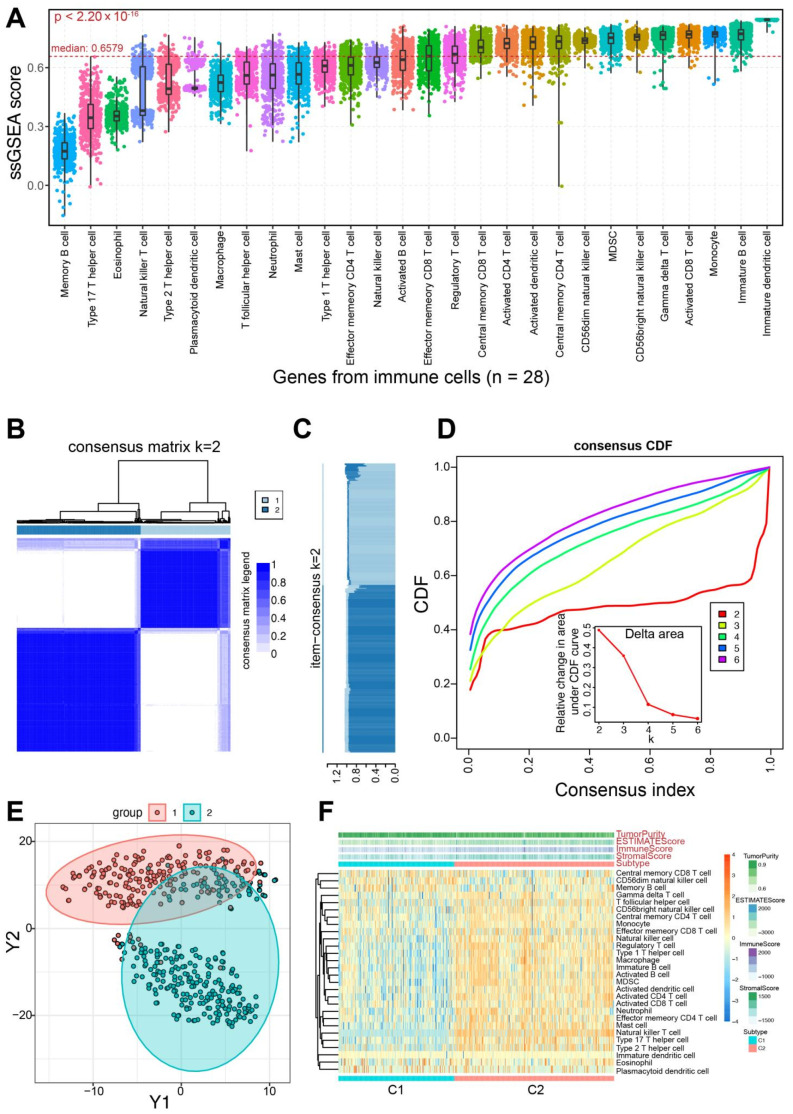
Identifying immune-based molecular subtypes in prostate cancer. (**A**). Distributions of ssGSEA scores are presented across different gene sets from 28 immune cells, and the total median value is highlighted (0.6579). The *p* value is estimated using a trend test. (**B**). The consensus score matrix for PRAD samples (k = 2) indicates that the two clusters can be divided. (**C**). The item-consensus analysis shows that k = 2 is an optimal selection. (**D**). Cumulative distribution function (CDF) curve of the consistency score shows that k = 2 is an optimal selection based on different subtype numbers (k = 2–6). Delta area plot of the relative change in area under CDF curve is also presented. (**E**). The two clusters can be distinguished based on ssGSEA scores using principal component analysis (PCA) of all samples. Each point indicates a patient, and different colors indicate the relevant subtypes. (**F**). A heatmap of immune characteristics based on ssGSEA scores shows the whole distributions in the two identified subtypes.

In order to identify subtypes in PRAD, 782 genes from 28 immune-associated gene sets were analyzed to estimate ssGSEA score in each sample using expression profiles (Figure 1A), and the median ssGSEA score was 0.6579 (the range was from −0.1541 to 0.8459). The ssGSEA scores showed significant difference based on different gene sets from different immune cells (*p* < 2.20 × 10^−16^), and genes in memory B cells were found to have a lower score (the median score was 0.1741) than others, while genes in immature dendritic cell showed the highest score (the median score was 0.8448). Then, based on the ssGSEA scores, k subtypes (2–6) could be divided, and the optimal number of clusters was defined k = 2 according to the consistency matrix heatmap and CDF curve (Figure 1B–D, Figures S1 and S2). Further analysis using t-SNE method also showed that two molecular subtypes were distinguished, including C1 and C2 subtypes (Figure 1E).

### 3.2. Immune Characteristics of the Two Identified Subtypes

**Figure 2:**
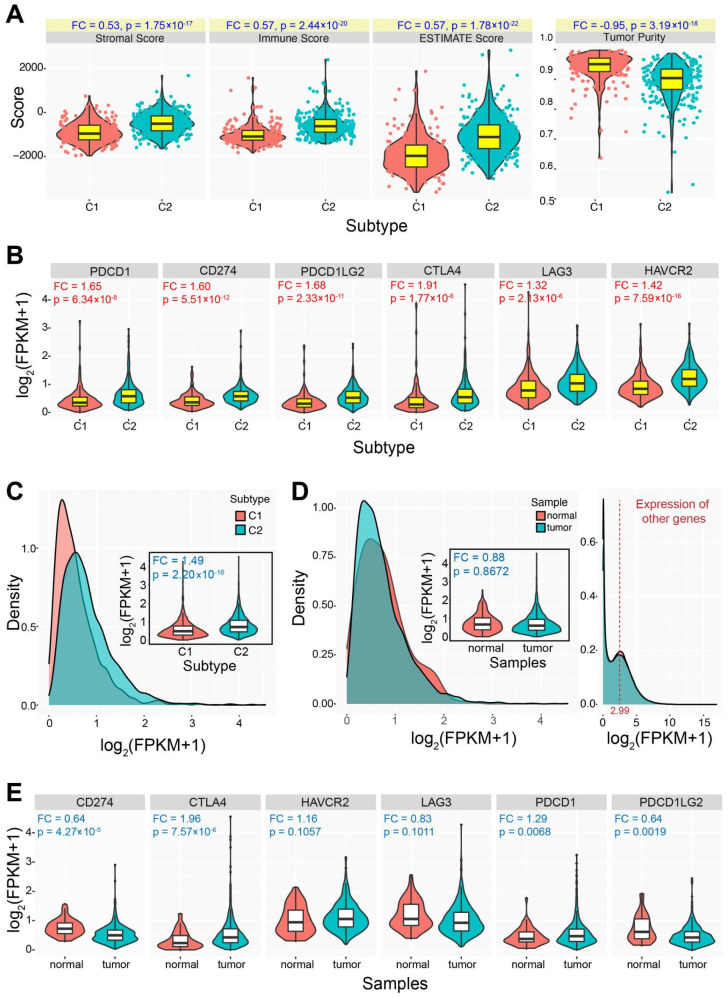
The distributions of immune-related features and genes between the two subtypes. (**A**). The distributions of immune-related features between the two subtypes. The fold change and *p* values are also presented for each feature. (**B**). Differential expression patterns of several immune checkpoint genes between the two subtypes. (**C**). The whole expression distributions of immune checkpoint genes between the two subtypes, and the fold change and *p* value are also presented. (**D**). The whole expression distributions of immune checkpoint genes between tumor and normal samples, and the fold change and *p* value are also presented. The distribution on the right indicates the expression distribution of other genes in PRAD, and the median value is 2.99 EUR. (**E**). The detailed expression patterns of immune checkpoint genes in PRAD, and the fold change and *p* value are also presented.

To estimate immune characteristics of the two identified subtypes, each was queried for the potential difference in different immune-related features using ESTIMATE. For genes in different cell types, higher ssGSEA scores in the C2 subtype could be detected with relevant gene sets in regulating T cell, macrophages, Th1 cell and other relevant immune processes (Figure 1F). The C2 subtype showed higher levels of stromal (FC = 0.53, *p* = 1.75 × 10^−17^), immune (FC = 0.57, *p* = 2.44 × 10^−20^) and ESTIMATE (FC = 0.57, *p* = 1.78 × 10^−22^) scores than those in the C1 subtype, indicating that the C2 had higher degree of infiltration than that in the C1 subtype. Further, the C2 showed lower level of tumor purity than that in the C1 (FC = −0.95, *p* = 3.19 × 10^−18^) (Figure 2A), implying that the lower level of tumor purity was relevant with the higher degree of immune infiltration. 

**Figure 3:**
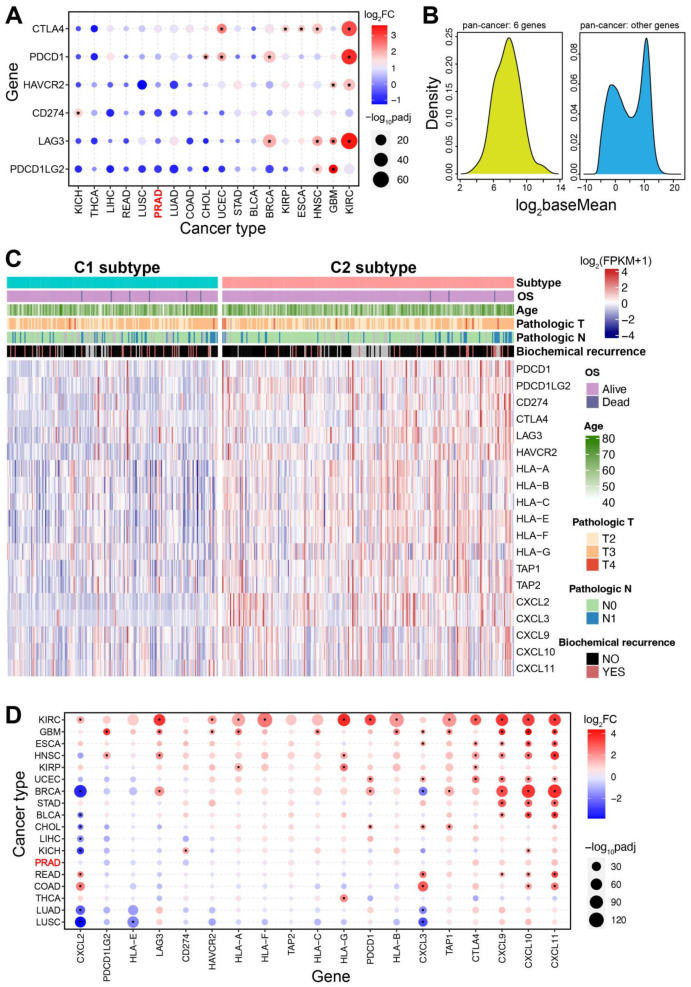
Pan-cancer expression analysis of the immune-related genes. (**A**). A pan-cancer expression analysis of immune checkpoint genes indicates diverse expression patterns across cancers. (**B**). The expression distributions of six immune checkpoint genes and other genes demonstrate the similar expression levels. The red word “PRAD” in Figure 3B is the main cancer type in this study. (**C**). A heatmap shows expression distributions of the 28 immune gene sets between the two subtypes. (**D**). A pan-cancer expression analysis of related immune genes in (**C**) shows diverse expression patterns across cancers.

Six genes (including PDCD1, CD274, PDCD1LG2, CTLA4, LAG3, and HAVCR2) associated with immune checkpoint were used to understand expression divergence between different subtypes. Genes in the C2 subtype were prone to have higher expression levels than those in the C1 subtype (FC ≥ 1.32, *p* ≤ 2.13 × 10^−6^ for all genes) (Figure 2B,C). The expression distributions showed the obvious expression divergence between the C1 and C2 subtypes, and the total expression fold change was 1.49 (*p* = 2.20 × 10^−16^) (Figure 2C). Although the expression difference could be detected between the two subtypes, they were always more stably expressed in tumor samples than those in normal samples (the total fold change was 0.88, *p* = 0.8672) (Figure 2D,E), and only CTLA4 showed higher expression in tumor samples (FC = 1.96, *p* = 7.57 × 10^−6^). Compared to expression distribution of all other human genes (the median log_2_(FPKM + 1) value was 2.99), these six genes had lower expression levels (Figure 2D), which demonstrated that the involved genes were not active enough in tumor samples compared to those in normal samples. The relative higher expression patterns in the C2 subtype indicated the potential divergence between the two classified subtypes. To further verify the expression pattens of the six genes associated with immune checkpoint, pan-cancer expression analysis was performed. As expected, these genes were stably expressed in most cancers, and some of them were found significantly up-regulated in several cancers, especially in kidney renal clear cell carcinoma (KIRC) (Figure 3A). CTLA4, PDCD1, and LAG3 were significantly up-regulated in 4–5 cancers, while all of them were not dysregulated in PRAD. Based on the total samples across different cancers, the six genes were relatively abundantly expressed compared to other genes (Figure 3B), whereas they showed lower expression levels in PRAD. The potential expression divergence among cancers implied two expression patterns in diverse cancers, which might contribute to relevant function. 

Further, to understand the potential differences between immune checkpoint, chemokine, and major histocompatibility complex (MHC) in the characterized subtypes in PRAD, a series of relevant genes were collected. Significant differences could be detected between the two subtypes (Figure 3C), although it was similar in some clinical features. Similar to the six core checkpoint genes, these genes also showed various expression patterns across cancers, while they were specifically stably expressed in PRAD (Figure 3D). Interestingly, most genes in KIRC and glioblastoma multiforme (GBM) were significantly up-regulated, and the phenomenon of over-expression was prominent in cancers. However, these genes did not show dysregulation expression in PRAD, and it was the only type of cancer that did not exhibit abnormally expressed immune-relevant genes. The specific expression pattern of stable expression implied the potential importance of immune-related roles, or immune-related genes would not have been affected by the pathological processes in prostate cancer. 

### 3.3. Differences in Drug Sensitivity between the Two Subtypes

**Figure 4:**
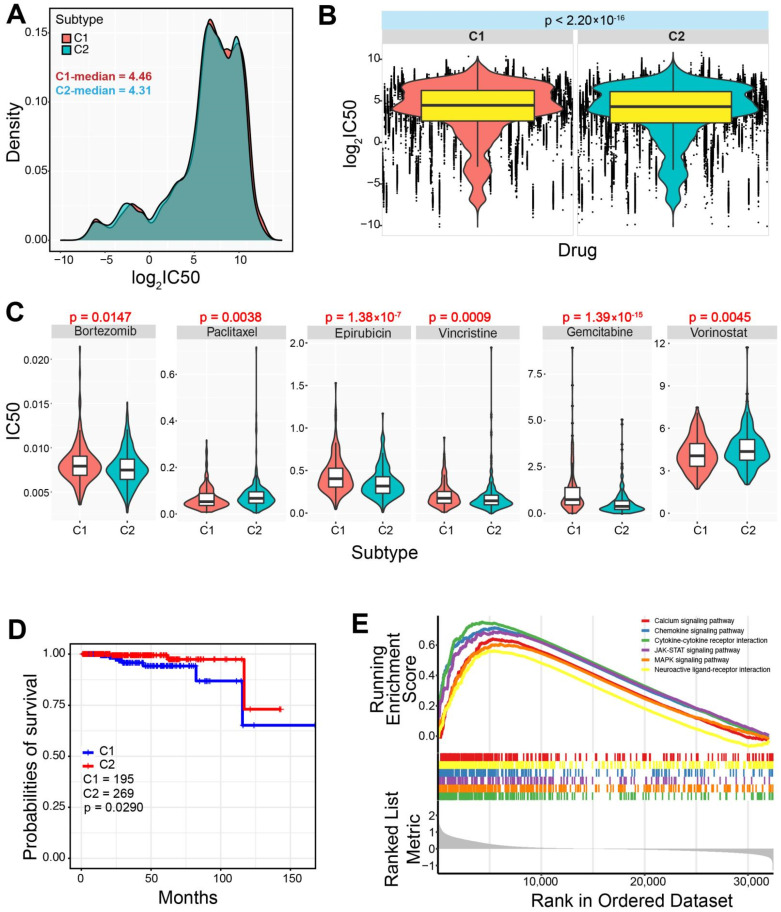
Drug response analysis between the two screened subtypes in PRAD. (**A**). The distributions of the IC50 values between the two subtypes, and the median values are also presented. (**B**). The detailed distributions of the IC50 values between the two subtypes (*p* < 2.2 × 10^−16^ is estimated using the trend test). (**C**). Several drugs show significant differences of IC50 values between the two subtypes. (**D**). Survival analysis shows distinct survival outcomes between the two subtypes. (**E**). Gene set enrichment analysis (GSEA) of the dysregulated genes in the C2 compared with those in the C1 subtype indicates significantly enriched several biological pathways.

The two subtypes showed a significant difference in drug sensitivity based on all drugs (Figure 4A,B, *p* < 2.20 × 10^−16^), and the C1 subtype showed a higher IC50 value than that of the C2 subtype. For example, some drugs (such as Bortezomib, Paclitaxel, Epirubicin, Vincristine, Gemcitabine, and Vorinostat, as well as the six drugs which were potentially effective for PRAD patients) were found to have significant differences in IC50 between the two subtypes (Figure 4C), implying that the two identified subtypes had diverse drug sensitivity. The potential difference indicated that patients with different subtypes could be treated with corresponding drugs. 

### 3.4. Difference of Cancer Prognosis between the Two Subtypes

A significantly increased chance of survival in the C2 subtype could be found compared to the C1 subtype (*p* = 0.0290) (Figure 4D), indicating that patients with the C2 subtype may have a better prognosis. In order to further understand the potential biological roles of immune-related genes in the C2 subtype, differentially expressed genes were obtained based on the C1 subtype. Abnormally expressed genes in the C2 subtype contributed to multiple biological processes, mainly including the calcium signaling pathway, chemokine signaling pathway, JAK−STAT signaling pathway, MAPK signaling pathway, etc. (Figure 4E). These potential contributions might lead to better prognosis and survival rate in the C2 subtype. 

**Figure 5:**
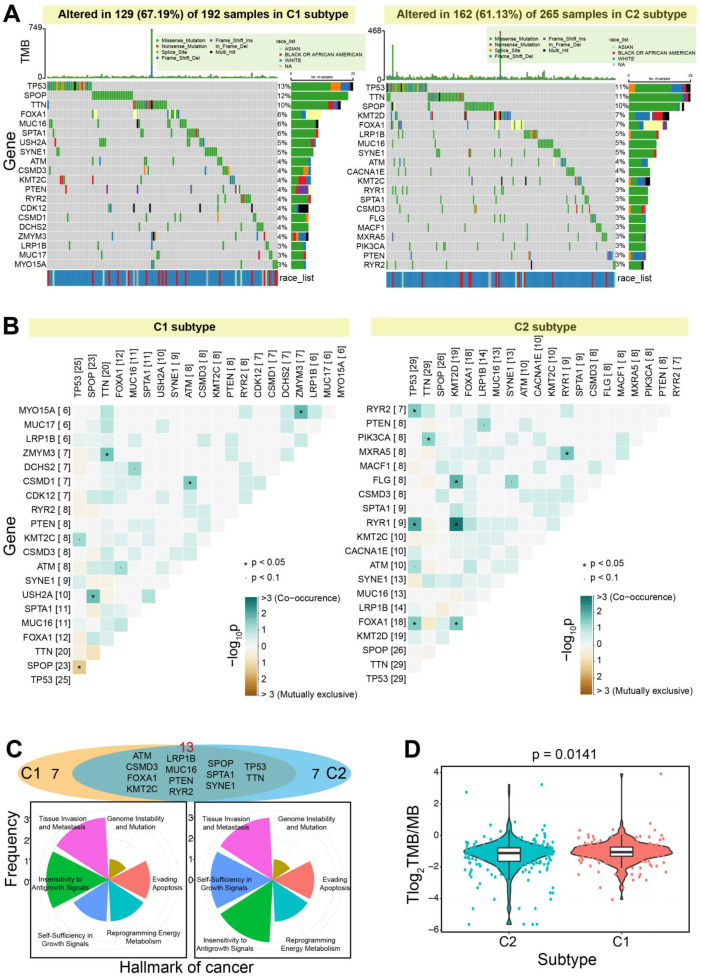
Somatic mutation landscapes of the two subtypes. (**A**). Somatic mutation landscapes of the two subtypes based on the top 20 genes with higher mutation frequencies, indicating the differences between the two subtypes. (**B**). Heatmaps show somatic interactions between the top 20 genes. (**C**). The gene distributions of the top 20 genes between the two subtypes and their potential contributions in hallmark of cancer. (**D**). The significant difference of the tumor mutational burden can be found between the two subtypes.

Further, the two subtypes also showed various mutation landscapes (Figure 5). Based on the top 20 genes with the highest mutation frequency, 67.19% of mutation frequency was found in the C1 subtype, but only 61.13% was detected in the C2 subtype. TP53 was identified with the highest mutation frequency in the two subtypes, and the most common type of mutation was missense mutation. Indeed, TP53 mutations are correlated with clinical outcomes in cancer, and it has clinical value in the diagnosis, prognosis, and treatment of cancer [30]. TP53 was detected concurrently with mutation with RYR1 in the C2 subtype, and was found to be mutually exclusive with mutation with SPOP in the C1 subtype (Figure 5B). Based on the top 20 genes in each subtype, the concurrency was more frequent than the mutually exclusive mutations. The correlations of mutations in genes might imply the potential functional correlations. Only 13 common genes were detected between the two subtypes, and they contributed to the hallmark of cancer with similar distributions (Figure 5C). The tumor mutational burden was diverged between the two subtypes (*p* = 0.0141) (Figure 5D), despite the total distributions being similar. Moreover, to understand the potential effects of clinical characteristics between the two subtypes, statistical analysis was performed on some clinical features. No significant differences in clinical characteristics were found between the two subtypes in TCGA PRAD cohort (FDR > 0.05, Table S1). 

### 3.5. Construction of Immune-Related Prognostic Model 

**Figure 6:**
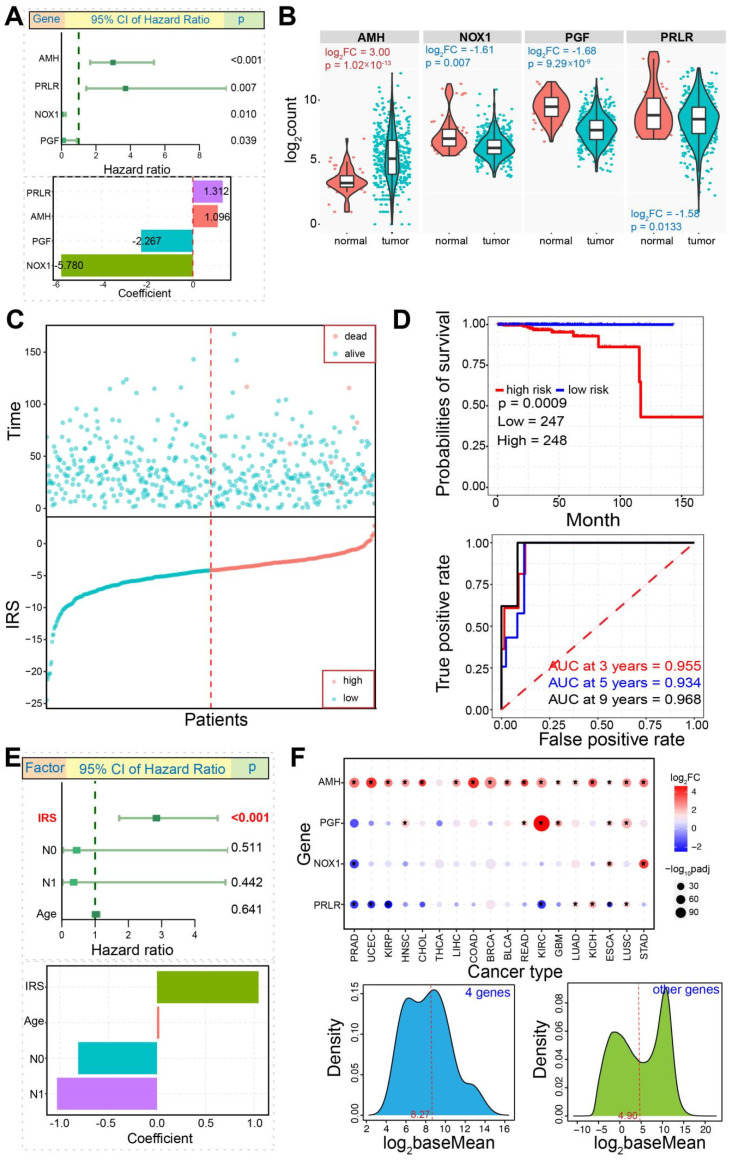
Four immune-related dysregulated genes are finally screened. (**A**). A graph presents distributions of hazard ratios, and corresponding coefficients for four selected genes are also presented. (**B**). AMH is significantly up-regulated, while other genes are significantly down-regulated in tumor samples. The detailed log_2_FC and *p* values are also presented. (**C**). Distributions of risk scores and survival times in patients show that all dead patients are clustered together. (**D**). Distinct survival difference can be found between high risk and low risk groups, and the ROC curve shows better performance at each cutoff point. (**E**). A graph presents distributions of hazard ratios, and corresponding coefficients for IRS and other factors are also presented. (**F**). A pan-cancer expression analysis of four screened key genes shows dynamic expression across diverse tissues. The relative expression distributions based on baseMean value estimated by DESeq2 algorithm are presented, and expression distributions of other genes are also presented. The expression median values are highlighted using a red dotted line. The expression distributions imply a higher expression trend of the four crucial genes compared to other genes.

In order to construct an immune-related prognostic model, analysis was performed on all immune-related genes to obtain abnormally expressed genes in PRAD. A total of 253 genes, including 138 up-regulated genes and 115 down-regulated genes, were firstly subjected to Cox analysis to screen 16 candidate immune genes associated with PRAD based on the Wald test *p* < 0.10; all of these genes showed potential prognostic value. Then, they were incorporated into a multivariate Cox proportional hazards regression model to further screen crucial genes associated with PRAD. We finally screened four crucial immune-related genes (Figure 6A,B), including three down-regulated genes (PRLR, HR = *p* = 0.007; NOX1, *p* = 0.010; PGF, *p* = 0.039) and one up-regulated gene (AMH, *p* < 0.001). Of these, three down-regulated genes showed abundant expressions than that in AMH, and NOX1 and PGF presented relative concentrated expression patterns (Figure 6B). All of them contributed to multiple Kyoto encyclopedia of genes and genomes (KEGG) pathways, for example, pathways in cancer, TGF-β signaling pathway, JAK/STAT signaling pathway, etc., indicating these screened genes had important biological roles in multiple biological processes. Further, three up-regulated genes, PRLR, NOX1, and PGF, also contributed to several hallmarks of cancer, mainly including insensitivity to antigrowth signals, self-sufficiency in growth signals, and evading apoptosis, indicating their potential roles in tumorigenesis. 

According to the potential contributions in multiple biological processes and tumorigenesis, the four screened genes were used to construct a survival model according to: IRS = 1.096 × AMH + 1.312 × PRLR + (−5.780) × NOX1 + (−2.267) × PGF. Thus, each patient’s immune risk score was calculated, and then each was identified as part of the high-risk or low-risk group according to the median value (Figure 6C). As expected, several dead patients were clustered in the high-risk group, and all of them showed higher risk scores that implied poor prognosis. In order to estimate the prognostic effect of the model, survival analysis was performed. Significant differences could be found between the high-risk and low-risk groups (*p =* 0.0009, Figure 6D), and the low-risk group had a better chance of survival than those in the high-risk group. The AUC was 0.968 at 9 years (0.955 at 3 years and 0.934 at 5 years) (Figure 6D), implying that the developed model had high accuracy and potential diagnostic value. Simultaneously, some factors were also performed using multivariate Cox regression analysis as well as IRS, including tumor stages and age (Figure 6E). IRS was the factor that had the greatest impact on prognosis compared to others (*p* < 0.001), which indicated that IRS could be an independent prognostic factor. These findings could provide additional data for prognostic risk assessment in prostate cancer.

### 3.6. The Two Immune-Based Subtypes Show Potential Application in Other Cancers

**Figure 7:**
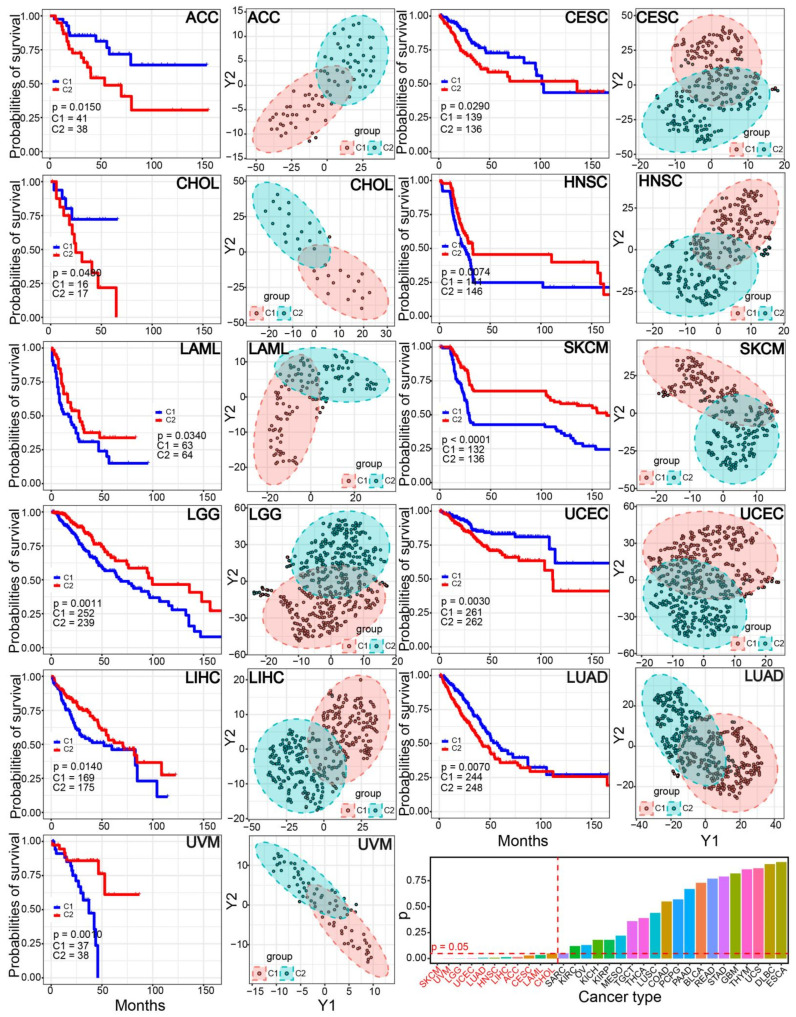
A pan-cancer analysis shows the two relatively independent clusters and potential prognostic values in 11 cancer types. The *p* values of survival analysis are also presented, and 11 cancers are highlighted.

In order to understand the potential application of the two characterized immune-based subtypes, a pan-cancer analysis was further performed. In 31 cancer types, many cancers could be divided into the two relatively independent C1 and C2 subtypes, implying the identified two subtypes also had common features in diverse cancer types (Figure 7). The significant survival differences were detected in 11 cancer types (35.48%), especially in skin cutaneous melanoma (SKCM), uveal melanoma (UVM) and brain lower grade glioma (LGG) (*p* < 0.0011), implying the potential prognostic values of the immune-based subtypes in other cancers.

## 4. Discussion

Identification of molecular subtypes in cancers contributes to more accurate prognostic assessment due to substantial phenotypic and molecular heterogeneity among patients, and simultaneously is an important basis for individualized therapy and precision medicine. Studies about molecular subtypes have been widely concerned with diverse cancer types, such as breast cancer [31,32], colon cancer [33,34] and gastric cancer [35], which provide potentially crucial data for diagnosis and treatment. In prostate cancer, via a comprehensive molecular analysis, seven major molecular subtypes are characterized by the Cancer Genome Atlas Research Network, TCGA-PRAD project [36], and relevant studies are also carried out to provide more data in cancer treatment [37,38,39]. Based on the area of cancer immunotherapy in prostate cancer [40,41], immune-based molecular subtypes are necessary in that they can provide important references for further immune therapy. Herein, we aim to identify immune-based molecular subtypes that may provide more detailed information for cancer therapy, and simultaneously we also develop an immune-related prognostic model to assess cancer prognosis. 

The two immune-based molecular subtypes are characterized based on integrating immune-related genes, and the two subtypes show significant differences in immune characteristics (Figure 2). The C1 subtype is identified as an immune “desert” type, while the C2 subtype is an immune-infiltrating type. Compared to the C1 subtype, the C2 subtype demonstrated a higher level of immune infiltration and lower tumor purity. Genes associated with the immune checkpoint show diverged expression patterns between the two subtypes, and genes in the C2 subtype are prone to have higher expression levels than those in the C1 subtype. However, these genes may be more stably expressed in tumor samples compared with expression in normal samples, and the total expressions are relatively low. Similarly, the two subtypes also show the potential difference of chemokine and MHC, and all of the involved relevant genes are stably expressed in prostate cancer despite the fact that some of them are dysregulated in other cancers (Figure 3). Furthermore, the two subtypes also show a significant difference in drug sensitivity and survival rate (Figure 4). Specifically, the C2 subtype presents a better survival rate than that in the C1 subtype, showing the potential contribution in cancer treatment. Based on the C1 subtype, the dysregulated genes in the C2 subtype are enriched multiple pathways, and dysregulation of these pathways greatly contributes to the occurrence and development of cancers, and they are important in cancer therapy [42,43,44,45]. Moreover, diverse mutation landscapes are found between the two subtypes (Figure 5), and some of the top genes are involved in the hallmarks of cancer that imply the potential roles in tumorigenesis. Furthermore, the identified immune-based subtypes also show certain prognostic values in other cancers, indicating the potential application in other cancers (Figure 7). All of these results indicate that the two identified immune-based molecular types have different characteristics, which further verifies the potential clinical application in immune therapy. 

Moreover, to enrich the prognostic model in prostate cancer treatment, we also construct an immune-related prognostic model based on dysregulated immune-related genes. A total of four genes, including AMH, PRLR, NOX1, and PGF, are screened as predicators of the prognostic model (Figure 6). Of these, PRLR has been implicated in the pathology of breast and prostate cancer [46,47], NOX1 expression may be increased in prostate cancer with an important role in angiogenesis, cell growth, and tumor pathogenesis [48,49], and both AMH and PGF are reported as crucial genes in tumorigenesis [50,51,52]. Based on their potential biological roles and contributions in cancer, the developed immune-related prognostic model is sensitive and effective (AUC = 0.968, and the survival model is an independent prognostic factor), which would provide additional immune-based information for early risk appraisal and treatment management in prostate cancer. 

Taken together, the two immune-based molecular subtypes are identified with different characteristics, which clearly describe the heterogeneity of diverse immune microenvironments in patients with prostate cancer. Simultaneously, a prognostic model based on four immune-related dysregulated genes is developed, which may provide a robust and useful model from immune level for the individualized treatment plan and health resource utilization. 

## Data Availability

The data are available upon request.

## Supplementary Materials

The following supporting information can be downloaded at https://www.mdpi.com/article/10.3390/genes13061087/s1, Figure S1. The consensus score matrixes for PRAD samples based on different selection of k value (k = 3–6). Figure S2. The item-consensus based on different selection of k value (k = 3–6). Table S1. Comparison analysis of some clinical characteristics in TCGA PRAD cohort.

## Abbreviation Lists of Involved Cancers in TCGA

BLCA, bladder urothelial carcinoma; BRCA, breast invasive carcinoma; CHOL, cholangiocarcinoma; COAD, colon adenocarcinoma; ESCA, esophageal carcinoma; GBM, glioblastoma multiforme; HNSC, head and neck squamous cell carcinoma; KICH, kidney chromophobe; KIRC, Kidney renal clear cell carcinoma; KIRP, kidney renal papillary cell carcinoma; LIHC, liver hepatocellular carcinoma; LUAD, lung adenocarcinoma; LUSC, lung squamous cell carcinoma; PRAD, prostate adenocarcinoma; READ, rectum adenocarcinoma; STAD, stomach adenocarcinoma; THCA, thyroid carcinoma; UCEC, uterine corpus endometrial carcinoma.
