# Supplementary material for: Characterization of Immune-Based Molecular Subtypes and Prognostic Model in Prostate Adenocarcinoma
Source: Genes (Basel). 2022 Jun 18;13(6):1087. doi: 10.3390/genes13061087 (PMC9223199; doi:10.3390/genes13061087)
Supplement: Supplementary file 1 [file genes-13-01087-s001.zip › Figure-S1.pdf]

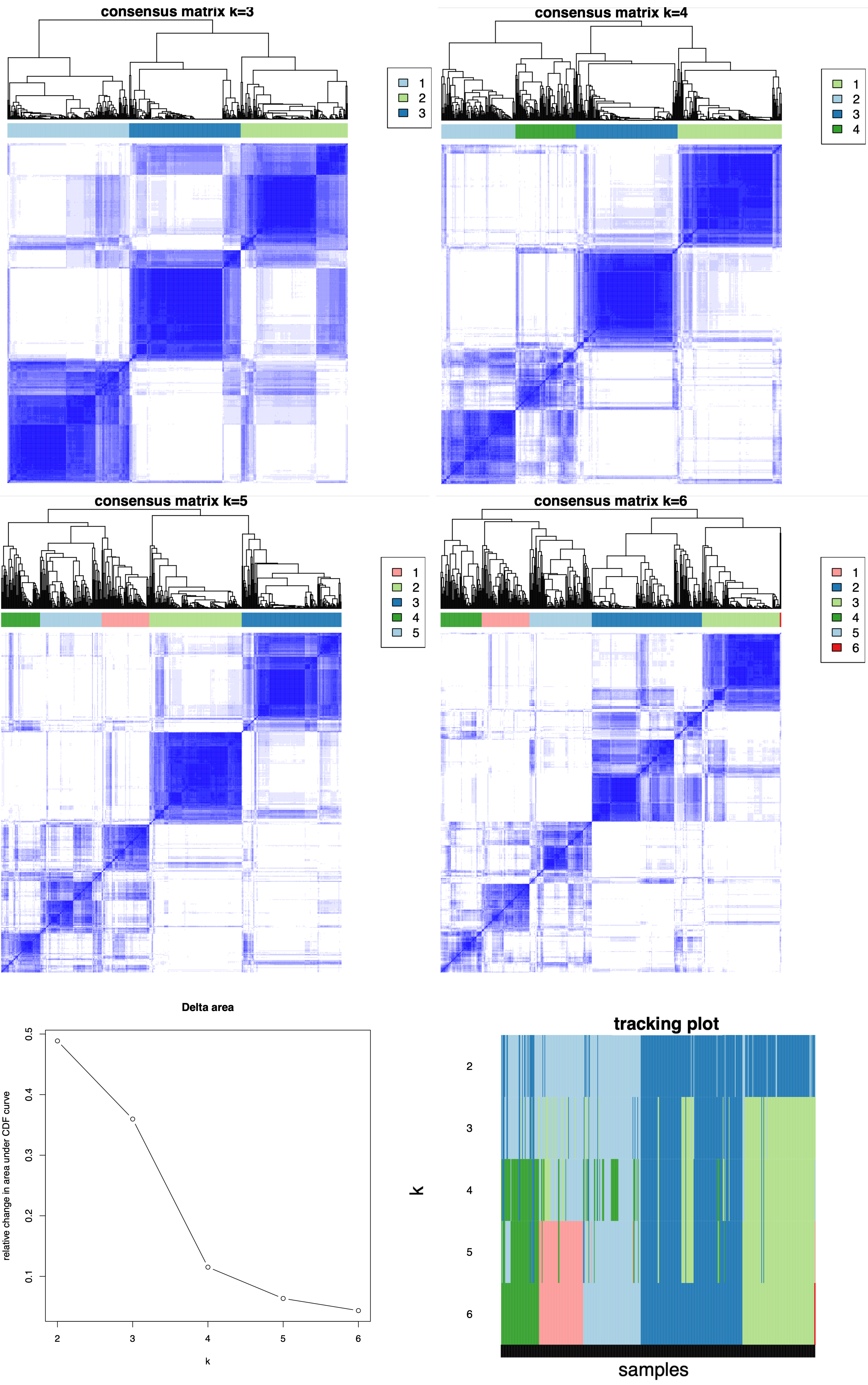
consensus matrix k=3
1
2
3
consensus matrix k=4
1
2
3
4
consensus matrix k=5
1
2
3
4
5
consensus matrix k=6
1
2
3
4
5
6
Delta area
relative change in area under CDF curve
0.1
0.2
0.3
0.4
0.5
2
3
4
5
6
k
tracking plot
k
2
3
4
5
6
samples
