# Supplementary material for: Characterization of Immune-Based Molecular Subtypes and Prognostic Model in Prostate Adenocarcinoma
Source: Genes (Basel). 2022 Jun 18;13(6):1087. doi: 10.3390/genes13061087 (PMC9223199; doi:10.3390/genes13061087)
Supplement: Supplementary file 1 [file genes-13-01087-s001.zip › Table S1.pdf]

Table S1. Comparison analysis of some clinical characteristics in TCGA PRAD cohort.

Parameter		Immune subtype (n, %)		P value	FDR
		C1 subtype (n=195)	C2 subtype (n=269)		
Age	≤65 years	59 (30.26)	74 (27.51)	0.5298	0.6055
	>65 years	136 (69.74)	149 (55.39)		
clinical_M	M0-1	177 (90.77)	251 (93.31)	0.3798	0.5064
	missing	18 (9.23)	18 (6.69)		
clinical_T	T1	65 (33.33)	95 (35.32)	0.3144	0.5030
	T2	69 (35.39)	97 (36.06)		
	T3-T4	28 (14.36)	24 (8.92)		
	NA	33 (16.92)	53 (19.70)		
pathologic_N	N0	122 (62.56)	201 (74.72)	0.0179	0.1432
	N1	38 (19.49)	37 (13.75)		
	missing	35 (17.95)	31 (11.52)		
pathologic_T	T2	67 (32.36)	108 (40.15)	0.1695	0.3506
	T3	124 (63.59)	150 (55.76)		
	T4	4 (2.05)	7 (2.60)		
	missing	0 (0)	4 (1.49)		
gleason_score	6	20 (10.26)	23 (8.55)	0.0394	0.1576
	7	84 (43.08)	148 (55.02)		
	8-10	91 (46.67)	98 (36.43)		
psa_value	<5.0	161 (94.15)	230 (95.04)	0.9382	0.9382
	5.0-9.9	3 (1.75)	4 (1.65)		
	>=10	7 (4.09)	8 (3.31)		
vital_status	Living	189(96.92)	266(98.88)	0.1753	0.3506
	Dead	6(3.08)	3(1.12)		

M, metastasis; T, tumor, N, lymph node. P values were obtained by Fisher's exact test, and FDR values were correctly by the Benjamini-Hochberg method.
