# Supplementary figures and images for: Characterization of Immune-Based Molecular Subtypes and Prognostic Model in Prostate Adenocarcinoma
Source: Genes (Basel). 2022 Jun 18;13(6):1087. doi: 10.3390/genes13061087 (PMC9223199; doi:10.3390/genes13061087)

item-consensus k=3

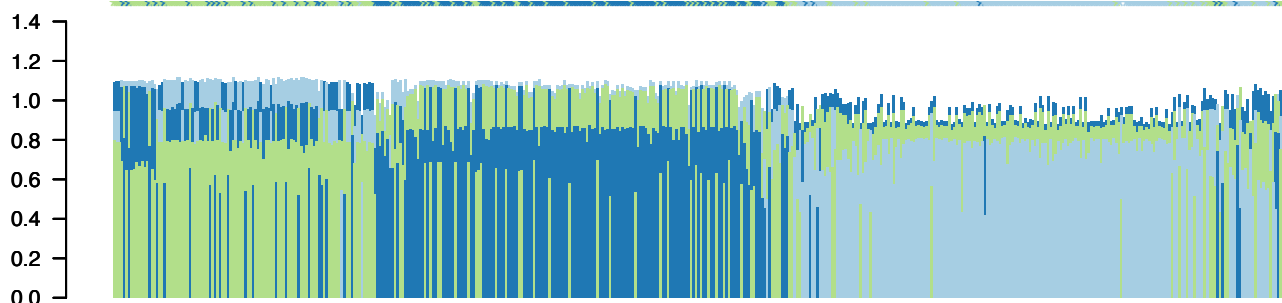

item-consensus k=4

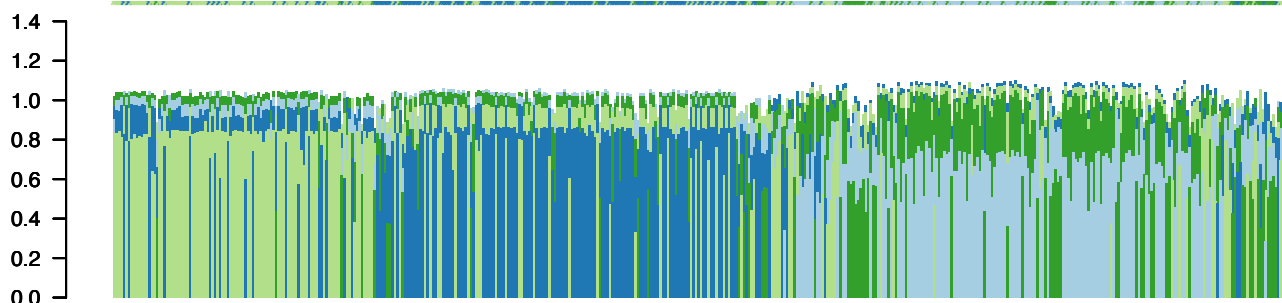

item-consensus k=5

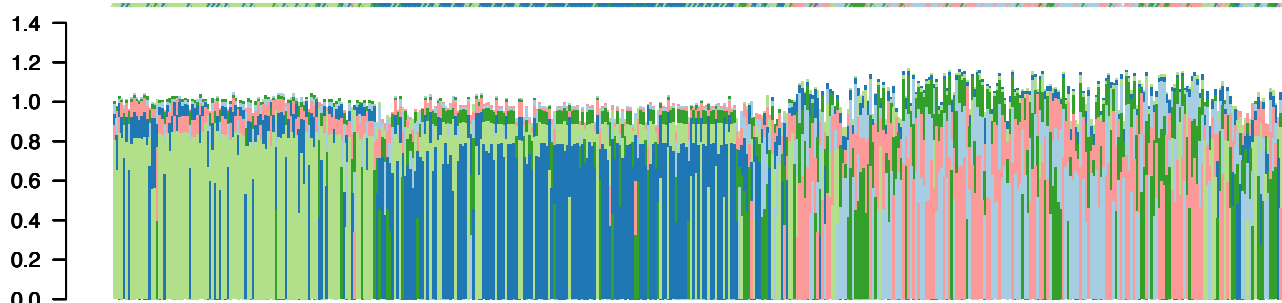

item-consensus k=6

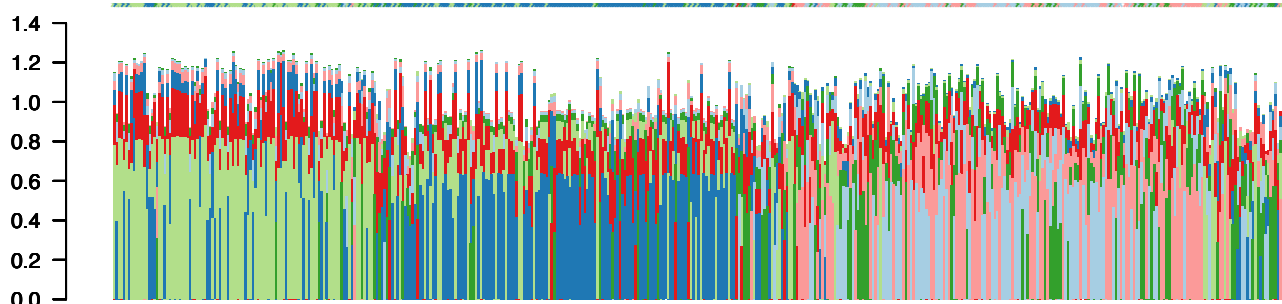

cluster-consensus

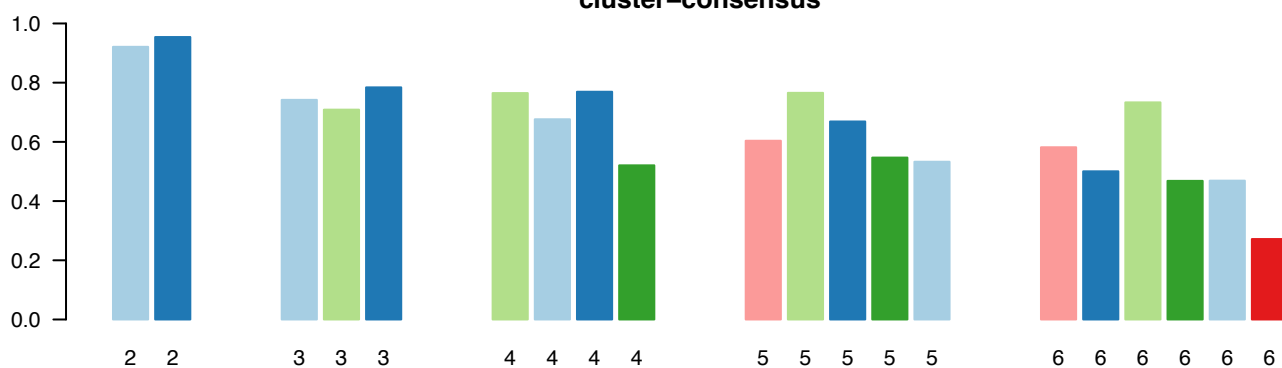

Supplement: Supplementary file 1 [file genes-13-01087-s001.zip › Figure-S2.pdf]
